# Myocardial Blush Grade Predicts Postoperative Atrial Fibrillation following Mitral Valve Replacement: A Novel Perspective

**DOI:** 10.3390/jcdd10070275

**Published:** 2023-06-29

**Authors:** Ömer Faruk Çiçek, Kerim Esenboğa, Muhammed Ulvi Yalçın, Mustafa Serkan Durdu, Bülent Behlül Altunkeser, Mustafa Büyükateş

**Affiliations:** 1 Department of Cardiovascular Surgery, Medical Faculty, Selçuk University, Konya 42250, Turkey; 2 Department of Cardiology, Medical Faculty, Ankara University, Ankara 06590, Turkey; 3 Department of Cardiology, Medical Faculty, Selçuk University, Konya 42250, Turkey; 4 Department of Cardiovascular Surgery, Medical Faculty, Ankara University, Ankara 06590, Turkey

**Keywords:** atrial fibrillation, mitral valve replacement, myocardial blush grading

## Abstract

**Background**: Atrial fibrillation (AF) remains the most common arrhythmia following mitral valve surgery. Although numerous clinical and laboratory indicators and possible mechanisms of postoperative AF (PoAF) have been described, the role of microvascular dysfunction in pathogenesis has not been assessed. We aimed to evaluate the association between microvascular dysfunction and PoAF in patients undergoing isolated mitral valve replacement. **Methods**: 188 patients undergoing mitral valve replacement were included in this retrospective study. Demographic characteristics of the patients were recorded. Angiographic assessment of microvascular perfusion was performed using the myocardial blush grading technique for each patient. Univariate and multivariate logistic regression analyses were utilized to determine predictors of PoAF. **Results**: Of 188 patients (56.69 ± 8.9 years, 39.4% male) who underwent mitral valve replacement, 64 (34%) patients developed PoAF. In the PoAF group, a lower basal hemoglobin level (12.64 ± 0.89 vs. 14.46 ± 0.91 g/dL; *p* < 0.001), a higher left atrial diameter [5.9 (5.2–6.47) vs. 4.9 (4.5–5.8) cm; *p* < 0.001], and a lower total blush score (TBS) (8.33 ± 0.84 vs. 8.9 ± 0.31; *p* < 0.001) were detected. Multivariate logistic regression analysis revealed that preoperative LA diameter (OR:2.057; 95% CI: 1.166–3.63; *p* = 0.013), preoperative hemoglobin (OR:0.12; 95% CI: 0.058–0.245; *p* < 0.001), and abnormal TBS (OR:15.1; 95% CI: 1.602–142.339; *p* = 0.018) were independent predictors of PoAF. **Conclusions**: Our findings demonstrated that TBS at the preoperative period was an independent predictor of PoAF in patients undergoing isolated mitral valve replacement.

## 1. Introduction

Postoperative atrial fibrillation (PoAF) remains a well-known and frequently encountered arrhythmia following cardiac surgery [1,2]. The occurrence of PoAF results in substantially higher postoperative mortality and morbidity, involving increased risk of cerebrovascular accidents, thromboembolic events, infections, and renal injury [3,4,5,6]. Furthermore, it is not entirely safe to use anticoagulant and antiarrhythmic medications to treat PoAF episodes as they may subject patients to potential side effects. PoAF is also associated with an increased duration of hospital stay leading to significant financial cost [6]. Consequently, early recognition of patients at risk for PoAF and adequate preventive management is still a main challenge for cardiovascular surgeons in the current era.

The mechanisms underlying PoAF have not been clearly identified yet, and its pathophysiology varies from classic atrial fibrillation (AF) in many aspects [7,8]. Various complex pathophysiological processes have been proposed, including inflammation, electrolyte imbalance, adrenergic activation, and myocardial ischemia, mostly superimposed on the intrinsic atrial substrate, which is susceptible to AF induction and persistence [9]. Although it has been demonstrated that the severity of coronary artery disease was a good predictor of PoAF [10], data regarding the possible role of coronary microvascular dysfunction on PoAF pathophysiology are limited. 

The myocardial blush grading (MBG) method has been well established as a basic angiographic technique to identify the efficacy of coronary microcirculation [11]. To date, this method has been used to assess tissue perfusion in different coronary pathologies such as coronary artery ectasia, myocardial infarction, syndrome X, and coronary tortuosity [11,12,13,14]. Furthermore, it has been demonstrated that, in patients with AF but no epicardial coronary artery disease, baseline and stress myocardial perfusion are also considerably affected. Therefore, coronary microvascular dysfunction was accepted as a critical pathophysiological entity in patients with a history of AF [15]. However, in patients with normal epicardial coronary arteries undergoing mitral valve surgery, the evaluation of tissue-level perfusion in the catheterization laboratory using the MBG method to predict PoAF has not yet been performed.

In the current study, we aimed to investigate the effect of impaired microvascular perfusion on PoAF development following isolated mitral valve replacement, independent of other risk factors. We hypothesized that assessment of microvascular perfusion via MBG, a straightforward angiographic instrument, might provide incremental predictive data for risk estimation of PoAF beyond biological and clinical parameters.

## 2. Materials and Methods

### 2.1. Study Design

This study was designed as a double-center, non-randomized, observational, retrospective study. It was carried out to evaluate the relationship between preoperative MBG score and PoAF in patients undergoing isolated mitral valve surgery. The study was in compliance with the principles outlined in the Declaration of Helsinki and approved by the Local Ethics Committee of the Selçuk University Medical Faculty (approval number: 2020/202 and approval date: 13 May 2020). In consideration of the acquired approval from the ethics committee, consent was granted by both hospitals involved in the study for its execution (Ankara University Hospital: 32557014-604.01.02-E.16460 and Selçuk University Hospital: 30292447-619/).

**Figure 1:**
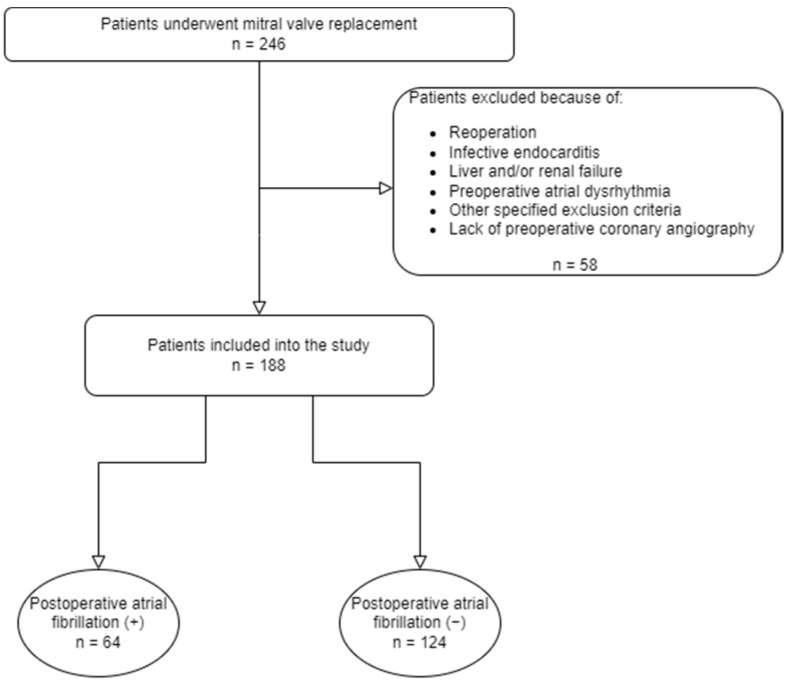
Flowchart of the study design.

The study population consisted of one hundred and eighty-eight patients scheduled for elective isolated mitral valve replacement for severe mitral valve pathology. Patients with a history or the presence of any atrioventricular conduction disorder or atrial dysrhythmia, hyperthyroidism, moderate-to-severe liver and renal failure, malignancy, pacemaker implantation, a contraindication to beta blockers, antiarrhythmic medication other than beta blockers, a history of previous cardiac operation, diabetes mellitus with late neurological complications, and more than 50% stenosis in any coronary territory, coronary spasm, coronary fistulae, and myocardial bridging evident on coronary angiogram were excluded. The study design is presented in the flowchart diagram (Figure 1).

Arterial blood pressure > 140/90 mmHg or receiving antihypertensive treatment was defined as hypertension. Diabetes mellitus (DM) was described as fasting plasma glucose ≥ 126 mg/dL or random plasma glucose ≥ 200 mg/dL plus diabetic symptoms or 2 h plasma glucose ≥ 200 mg/dL in the oral glucose tolerance test or HbA1C level ≥ 6.5. Two-dimensional echocardiographic assessment and laboratory tests were performed before surgery. All demographic and baseline clinical features of the patients were recorded.

### 2.2. Evaluation of Myocardial Blush Grading

Coronary angiography was consistently performed using the Judkins approach with 6F diagnostic catheters (Boston Scientific, Boston, MA, USA) in a standardized manner. Contrast injection was performed manually during the procedure. To be included in the study, the recordings had to be recorded until the venous phase, as required to evaluate MBG. In each patient, the best projection was preferred to determine the myocardial region of the coronary artery being examined. Two experienced interventional cardiologist who were blind to patients’ clinical data assessed the tissue-level perfusion using the MBG technique, as described: in [4] grade 0, no myocardial blush or contrast density; grade 1, minimal blush or contrast density; grade 2, moderate myocardial blush or contrast density, but less than that obtained in the same coronary territory of an age-matched and sex-matched control participant; grade 3, normal myocardial blush or contrast density, comparable with that obtained in the same coronary territory of an age-matched and sex-matched control participant. After evaluating microvascular perfusion using the MBG technique, the total blush score (TBS) was measured for both groups. TBS was calculated as the total amount of the blush grades of each coronary territory. Intraobserver and interobserver variability were calculated from a random selection of 40 coronary territories evaluated by reviewers.

### 2.3. Postoperative Atrial Fibrillation

PoAF has been described as a series of supraventricular beats with irregular R-R intervals that persist for 30 s or longer in the absence of P-waves. Continuous electrocardiography (ECG) monitoring in the intensive care unit was used to follow the subjects for the first 24 h postoperatively, while 12-lead ECG recording was checked regularly for rhythm follow-up on other days. When the subjects complained of angina, dyspnea, or palpitation, another 12-lead ECG was taken. Correction of electrolyte imbalance and appropriate fluid restitution were performed as required when PoAF occurred, followed by additional beta blockers and eventually amiodarone to provide medical conversion. 

### 2.4. Statistical Analysis

Statistical analyses were performed using the SPSS software package (version 25.0 for Windows; IBM; Armonk, NY, USA). The Kolmogorov–Smirnov test was used to identify the distribution of variables. The data were expressed as mean (±SD) for normally distributed variables and median with interquartile range (IQR) for skewed continuous variables. Categorical variables were expressed as percentages. The Student t-test or Mann–Whitney U test were used to compare continuous variables as appropriate. Fisher’s exact and continuity correction (Yate’s correction) tests were used to compare categorical variables. In order to facilitate the regression analysis, the MBG and TBS values were converted into a dichotomous variable. For MBG, a score of 3 was classified as normal for each coronary territory, while values below 3 were categorized as abnormal. Similarly, for TBS, values of 8 and 9 were considered normal, while values below this range were categorized as abnormal. This transformation allowed for a more streamlined evaluation of these variables within the regression analysis. The baseline variables which were found significant (*p* < 0.05) in the univariate analysis were included in a reverse multivariate regression model to determine the independent associates of PoAF. In this model, the least significant variable was progressively eliminated in each iteration until only the remaining variables exhibited statistical significance. The results of the model were reported as odds ratio (OR), 95% confidence interval, and *p* values. For all tests, a *p* value of <0.05 was considered statistically significant. 

## 3. Results

One hundred eighty-eight patients undergoing mitral valve replacement (56.69 ± 8.9 years, 39.4% male) were included in the current study. Baseline demographic, clinical, laboratory, and echocardiographic parameters of the study population are given in Table 1. PoAF was observed in 64 (34%) patients, while sinus rhythm persisted in 124 (66%) patients. Among baseline demographic, clinical, laboratory, and echocardiographic parameters, age was found to be higher (58.8 ± 6.07 vs. 55.6 ± 9.9 years; *p* = 0.007), left atrial (LA) diameter was found to be larger (5.9 (5.2–6.47) vs. 4.9 (4.5–5.8) cm; *p* < 0.001), and baseline hemoglobin level was found to be lower (12.64 ± 0.89 vs. 14.46 ± 0.91 g/dL; *p* < 0.001) in patients with PoAF (Table 1). In addition, cardiopulmonary by-pass (CPB) time (108.19 ± 14.17 vs. 100.15 ± 22.29 min; *p* = 0.003), cross-clamp time (78.81 ± 10.96 vs. 72.52 ± 17.9 min; *p* = 0.003), and postoperative hospital stay (7.2 ± 0.93 vs. 6.05 ± 0.83 days; *p* < 0.001) were found to be longer in patients with PoAF. 

A total of 192 coronary territories in patients with PoAF and a total of 372 coronary territories in the sinus rhythm group were assessed cautiously. Myocardial blush grades of each coronary territory are presented in Table 2.

Myocardial blush grades (presented as mean ± SD) in all coronary artery territories were significantly lower in patients with PoAF compared to the sinus rhythm group. Also, TBS was found to be lower (8.33 ± 0.84 vs. 8.9 ± 0.31; *p* < 0.001) in patients with PoAF (Table 3). 

Intraobserver and interobserver variabilities of myocardial blush grades are shown in Table 4. The MBG method demonstrated high levels of intraobserver and interobserver reproducibility, which were comparable to those reported in a previous study [11].

To assess the predictors of PoAF, univariate and multivariate logistic regression analyses were performed. Univariate logistic regression analysis showed that age, preoperative LA diameter, baseline hemoglobin, CPB time, cross-clamp time, abnormal MBG scores for LAD, Cx, RCA territories, and abnormal TBS were risk factors for PoAF (Table 5). Multivariate logistic regression analysis revealed that preoperative LA diameter (OR:2.057; 95% CI: 1.166–3.63; *p* = 0.013), baseline hemoglobin (OR:0.12; 95% CI: 0.058–0.245; *p* < 0.001), and abnormal TBS (OR:15.1; 95% CI: 1.602–142.339; *p* = 0.018) were independent predictors of PoAF (Table 6).

## 4. Discussion

Findings of the current study demonstrated that decreased TBS, increased left atrial diameter, and lower baseline hemoglobin levels were independent predictors of PoAF in patients undergoing isolated mitral valve replacement. These results established that assessment of microvascular perfusion preoperatively via the angiographic MBG method can be useful for prediction of PoAF in this patient group. To the best of our knowledge, this is the first study demonstrating the association between microvascular perfusion and PoAF following isolated mitral valve replacement. 

The risk of PoAF was documented to be higher in valvular and combined procedures involving coronary and valvular surgery than in coronary surgery alone. The PoAF rates were reported by Mariscalco et al. [16] as 22.9%, 39.8%, and 45.2% for isolated CABG, valve surgery, and combined procedures, respectively. Bramer et al. [17] identified the percentages of PoAF in men and women for isolated mitral valve surgery as 42.2% and 36.5%, respectively. In the postoperative phase after isolated mitral valve surgery, we observed a PoAF incidence of 34.8% in accordance with the previous reports, with a high rate of recovery to sinus rhythm following appropriate medical treatment.

The pathophysiology of AF development in the postoperative period is thought to be complex, consisting of several clinical and perioperative factors such as older age, male sex, past medical history of heart failure, chronic obstructive pulmonary disease, chronic kidney disease, diabetes mellitus, metabolic syndrome, obesity, high levels of brain natriuretic peptide, severe coronary artery disease, increased LA dimension, anemia, and blood transfusion preceding the surgery [18,19]. In terms of preoperative increased left atrial diameter and decreased hemoglobin levels, our results are consistent with those in the literature.

Left atrial diameter is an indirect measure of left ventricular filling pressure, which is frequently elevated in patients with mitral regurgitation. Indexed left atrial maximum volume was reported to be a robust, independent predictor of PoAF, even after adjustments for other biological and clinical parameters [20]. Osranek et al. [21] defined that the increased left atrial volume was associated with a five-fold increase in the PoAF risk, when the same cut-off value of 32 mL/m^2^ was used. In our study, although routine measurement of left atrial volume was not performed, the diameter of the left atrium was determined to be independently associated with PoAF. This finding adds to the notion that the left atrial structural remodeling had an impact on the occurrence of AF following mitral valve surgery.

Our findings revealed that decreased hemoglobin level was also independently associated with PoAF. Preoperative anemia has been demonstrated to independently increase postoperative mortality and morbidity along with the development of AF following heart surgery [22,23]. In the present study, an independent and inverse correlation was observed between preoperative hemoglobin levels and PoAF, consistent with previous reports. There may be several reasons for the association between preoperative anemia and development of PoAF in patients after mitral valve surgery. During the perioperative period, anemic patients are at higher risk for insufficient delivery of oxygen which may result in reduced tissue oxygenation and PoAF. Additionally, anemia is the most powerful indicator of hemodilution during CBP, and low levels of hematocrits are significantly related with adverse outcomes.

If contrast injection is adequately performed and the duration of cine angiography is sufficient, tissue level vasculature filling exhibits a “ground-glass” or an angiographic “blush” appearance. This “ground-glass” view can be used in the catheterization laboratory to evaluate microvascular filling visually and is thus a sign of microvascular perfusion disorder [24]. The MBG method is more feasible in comparison with other approaches such as nuclear testing, magnetic resonance imaging, contrast echocardiography, or positron emission tomography for rapid evaluation of coronary microcirculatory disorders during coronary angiography. TBS (sum of blush grades of each coronary territory) has been reported in clinical research to describe microvascular perfusion abnormalities in syndrome X and dilated cardiomyopathy [13,25]. Although clinical consequences of this index are unclear, observing reduced TBS in patients developing PoAF implies that perfusion at the tissue level in this patient population is compromised.

The association between microvascular dysfunction and the occurrence of PoAF has not been fully elucidated and is likely to be complex. Patients with AF and without significant lesions in epicardial coronary arteries may still exhibit clinical signs of ischemia, such as angina, ST segment depression, or elevated troponin levels, particularly at the beginning of arrhythmia, indicating the presence of either microvascular perfusion disorder or arrhythmia-induced coronary spasm [26]. Myocardial perfusion and perfusion reserve in patients with persistent AF and without epicardial coronary artery disease were assessed utilizing positron emission tomography (PET) and radioactively labeled water. Not only myocardial blood flow at rest but also adenosine-induced hyperemic flow was found to be significantly decreased compared to age- and risk-matched controls [27].

Indeed, it is not clear whether coronary microvascular dysfunction is a pathophysiological substrate associated with AF or whether it is a consequence of this arrhythmia. A study evaluating coronary flow reserve in patients with lone recurrent atrial fibrillation confirmed impaired perfusion of atrial myocardium and deterioration of coronary flow reserve in this patient population, suggesting microvascular dysfunction as an important pathophysiological entity. In this research, several pathoanatomic changes such as atrial fibrosis, vascular degeneration of atrial cells, and perivascular and interstitial amyloid deposition accounted for the findings of normal baseline coronary flow and decreased coronary flow at peak hyperemia, as well as diminished coronary flow reserve, due to functional and/or structural changes in the microvascular network [28,29,30]. Wijesurendra et al. [15] used advanced cardiac magnetic resonance techniques to determine myocardial perfusion at baseline and during peak hyperemia in patients with a diagnosis of AF and no significant epicardial coronary artery disease, and they reported that a reduction in stress myocardial blood flow in these patients was not a direct outcome of the arrhythmia itself but may imply an ongoing coronary endothelial dysfunction leading to impaired microvascular perfusion. Additionally, in the subgroup of patients with no history of recurrent AF, this difference also persisted after ablation, reflecting that microvascular dysfunction might be recognized as a potential pathophysiological substrate rather than as a direct result of arrhythmia itself. In our study, our primary objective was to assess the impact of global microvascular perfusion impairment, which affects both the ventricles and atria, on the development of atrial fibrillation in the postoperative period. Instead of focusing solely on the effect of direct ventricular ischemia, we aimed to investigate the broader influence of microvascular perfusion impairment on this arrhythmia. In accordance with our hypothesis, this study has highlighted that atrial fibrillation itself can lead to left ventricular and left atrial dysfunction through disrupting myocardial blood flow. However, findings evaluating microvascular perfusion indicated that even after ablation, myocardial blood flow did not improve significantly [15]. This suggests that microvascular perfusion disturbance, affecting both the ventricles and atria, plays a crucial role in the development of atrial fibrillation.

In agreement with the aforementioned studies, microvascular perfusion disorder might have played a critical role in the occurrence of PoAF, as a relevant pathophysiological mechanism, in our study. Additionally, considering the practicality and accessibility of the MBG score as a preoperative evaluation method for microvascular perfusion disturbance, which can occur in both the ventricles and atria, we believe it provides a viable approach for predicting postoperative atrial fibrillation. This is particularly relevant when compared to more sophisticated tests such as PET or cardiac magnetic resonance.

### Study Limitations

While MBG has been well defined and validated in previous studies, direct visual assessment of coronary microvascular perfusion and not measuring coronary flow reserve via Doppler guide wire in angiography as an invasive technique are two of the most important limitations of our study. Nonetheless, we performed an intraobserver and interobserver variability analysis to validate the correct assessment of myocardial perfusion. More advanced echocardiographic parameters, such as left atrial strain and indexed left atrial maximal volume, were not measured in the present study. On the other hand, the left atrial diameter is a simple parameter, widely available and measurable with good reproducibility in daily clinical practice. There are no documented long-term clinical results for the patients. Furthermore, ECGs of the patients were recorded once daily after intensive care unit follow-up, which could have led to missing some of the paroxysmal AF attacks.

Inflammation serves as a possible link between AF, endothelial dysfunction, and a decreased myocardial perfusion and perfusion reserve [31]. Since the potential relationship between inflammation and microvascular dysfunction is beyond the scope of this study, inflammatory markers such as hsCRP were not analyzed. The fact that not all mitral valve surgeries have been performed by the same operator or surgical team may constitute a confounding factor. Finally, the relatively small patient population can also be accepted as a limitation of our study.

## 5. Conclusions

In conclusion, our study demonstrates that reduced TBS at the preoperative period is associated with PoAF following isolated mitral valve replacement. The routine measurement of TBS preoperatively might be helpful for the prediction of PoAF in patients undergoing isolated mitral valve surgery enabling proactive measures to mitigate potential complications. During the postoperative follow-up of patients with low TBS values, diligent attention should be given to the occurrence of atrial fibrillation, as well as monitoring for hemodynamic and metabolic changes that may contribute to its facilitation. This comprehensive approach can provide timely recognition and appropriate management of atrial fibrillation, minimizing associated risks and optimizing patient outcomes. Whether the determination of this score can be added to routine evaluation for prediction of AF in patients undergoing mitral valve surgery is a matter that needs further investigation.

## Figures and Tables

**Table 1:** Baseline characteristics of the study group.

Variable	Study Population (*n* = 188)	PoAF (+) (*n* = 64)	PoAF (−) (*n* = 124)	*p* Value
Age (years)	56.69 ± 8.9	58.8 ± 6.07	55.6 ± 9.9	0.007
Male gender	74 (39.4)	26 (40.6)	48 (38.7)	0.799
BMI (kg/m^2^)	26.36 ± 3.54	26.29 ± 3.53	26.4 ± 3.56	0.831
Hypertension	43 (22.9)	19 (29.7)	24 (19.4)	0.157
Hyperlipidemia	23 (12.2)	9 (14.1)	14 (11.3)	0.753
Diabetes mellitus	25 (13.3)	11 (17.2)	14 (11.3)	0.367
COPD	40 (21.3)	16 (25)	24 (19.4)	0.479
Current smoking	22 (11.7)	9 (14.1)	13 (10.5)	0.628
Preoperative NYHA status	1.97 ± 0.58	2.02 ± 0.49	1.94 ± 0.63	0.387
Preoperative ACEi/ARB use	40 (21.3)	17 (26.6)	23 (18.5)	0.278
Preoperative statin use	22 (11.7)	9 (14.1)	13 (10.5)	0.628
Preoperative EF	56.5 (49–63.75)	55 (50–60)	60 (48–65)	0.34
Preoperative LVEDD	5.65 (5.3–6.1)	5.8 (5.4–6.2)	5.6 (5.2–6.1)	0.04
Preoperative LVESD	4.1 (3.8–4.4)	4.1 (4–4.5)	4 (3.73–4.3)	0.045
Preoperative SPAP (mmHg)	45 (38–53.5)	44.5 (35.25–50)	45 (38–54.75)	0.669
Preoperative LA diameter (cm)	5.15 (4.63–6.1)	5.9 (5.2–6.47)	4.9 (4.5–5.8)	<0.001
Preoperative WBC (×10^9^/L)	8.34 ± 1.65	8.49 ± 1.78	8.27 ± 1.59	0.393
Preoperative hemoglobin (g/dL)	13.84 ± 1.25	12.64 ± 0.89	14.46 ± 0.91	<0.001
Preoperative ALT (U/L)	41.5 (35–48)	43 (36–51)	41 (35–46.75)	0.15
Preoperative AST (U/L)	36 (31–44.75)	35 (31–43.7)	37 (31.25–45)	0.503
Preoperative urea (mg/dL)	35 (29–43)	35 (30.25–42.75)	35 (28.25–43)	0.705
Preoperative creatinine (mg/dL)	0.83 (0.76–0.9)	0.82 (0.76–0.86)	0.83 (0.77–0.98)	0.074
Preoperative TSH (mIU/L)	2.3 (1.66–2.8)	2.3 (1.53–2.6)	2.35 (1.7–3.1)	0.101
Operation type				
MVR	134 (71.3)	47 (73.4)	87 (70.2)	0.764
MVR + tricuspid annuloplasty	54 (28.7)	17 (26.6)	37 (29.8)	
Prosthetic valve size (mm)	29 (27–31)	29 (27–31)	29 (27–31)	0.088
CPB time (minutes)	102.89 ± 20.22	108.19 ± 14.17	100.15 ± 22.29	0.003
X-clamp time (minutes)	74.66 ± 16.13	78.81 ± 10.96	72.52 ± 17.9	0.003
Postoperative inotrope	35 (18.6)	14 (21.9)	21 (16.9)	0.531
Postop hospital length (days)	6.44 ± 1.02	7.2 ± 0.93	6.05 ± 0.83	<0.001

Data are presented as *n* (%), mean ± SD or median (IQR). ACEi, angiotensin-converting enzyme inhibitors; ALT, alanine transaminase; ARB, angiotensin receptor blockers; AST, aspartate aminotransferase; BMI, body mass index; COPD, chronic obstructive pulmonary disease; CPB, cardiopulmonary; EF, ejection fraction; LA, left atrium; LVEDD, left ventricular end-diastolic diameter; LVESD, left ventricular end-systolic diameter; MVR, mitral valve replacement; PoAF, postoperative atrial fibrillation; SPAP, systolic pulmonary artery pressure; TSH, thyroid stimulating hormone; WBC, white blood cells.

**Table 2:** Myocardial blush grades of all coronary arteries are presented as a categorical variable.

	PoAF (+) (*n* = 64)	PoAF (−) (*n* = 124)	*p* Value
Left anterior descending artery			
1	1 (1.6)	-	
2	14 (21.9)	3 (2.4)	<0.001
3	49 (76.6)	121 (97.6)	
Circumflex artery			
1	4 (6.3)	-	
2	8 (12.5)	4 (3.2)	<0.001
3	52 (81.3)	120 (96.8)	
Right coronary artery			
2	11 (17.2)	6 (4.8)	0.005
3	53 (82.8)	118 (95.2)	

Data are presented as *n* (%). PoAF, postoperative atrial fibrillation.

**Table 3:** Myocardial blush grades of all coronary arteries and total blush score in the study groups are presented as mean ± SD.

	Study Population (*n* = 188)	PoAF (+) (*n* = 64)	PoAF (−) (*n* = 124)	*p* Value
Left anterior descending artery	2.9 ± 0.32	2.75 ± 0.47	2.98 ± 0.15	<0.001
Circumflex artery	2.89 ± 0.37	2.75 ± 0.56	2.97 ± 0.18	0.004
Right coronary artery	2.91 ± 0.29	2.83 ± 0.38	2.95 ± 0.21	0.018
Total blush score	8.7 ± 0.61	8.33 ± 0.84	8.9 ± 0.31	<0.001

Data are presented as mean ± SD. PoAF, postoperative atrial fibrillation.

**Table 4:** Intraobserver and interobserver variabilities of myocardial blush grades.

			Difference		
	*n*	Agreement	MBG 1	MBG 2	MBG 3
Intraobserver variability	40	38 (95)	1 (2.5)	1 (2.5)	-
Interobserver variability	40	38 (95)	2 (5)	-	-

Data are presented as *n* (%). MBG, myocardial blush grade.

**Table 5:** Independent predictors of PoAF in univariate analyses.

Variable	Univariate Analysis		
	OR	95% CI	*p*
Age	1.042	(1.006–1.078)	0.021
Hypertension	1.759	(0.876–3.533)	0.112
Preoperative LVEDD	1.648	(0.968–2.806)	0.066
Preoperative LVESD	1.768	(0.992–3.15)	0.053
Preoperative LA diameter	2.758	(1.869–4.071)	<0.001
Preoperative hemoglobin	0.091	(0.045–0.185)	<0.001
CPB time	1.021	(1.005–1.037)	0.011
X-clamp time	1.025	(1.005–1.045)	0.012
Abnormal MBG score for LAD	12.347	(3.422–44.552)	<0.001
Abnormal MBG score for Cx	6.923	(2.133–22.473)	0.001
Abnormal MBG score for RCA	4.082	(1.434–11.62)	0.008
Abnormal total blush score	44.489	(5.758–343.728)	<0.001

CPB, cardiopulmonary; Cx, circumflex; LA, left atrium; LAD, left anterior descending; LVEDD, left ventricular end-diastolic diameter; LVESD, left ventricular end-systolic diameter; MBG, myocardial blush grade; RCA, right coronary artery.

**Table 6:** Independent predictors of PoAF in multivariate analyses.

Variable	Multivariate Analysis		
	OR	95% CI	*p*
Preoperative LA diameter	2.057	(1.166–3.63)	0.013
Preoperative hemoglobin	0.12	(0.058–0.245)	<0.001
CPB time	1.025	(0.996–1.055)	0.096
Abnormal total blush score	15.1	(1.602–142.339)	0.018

CPB, cardiopulmonary; LA, left atrium.

## Data Availability

Data of the study can be made available to interested parties upon reasonable request to the corresponding author, subject to the approval of the Ethics Board and the Hospital’s authorized committees.

## References

[1] Echahidi N., Pibarot P., O’Hara G., Mathieu P. (2008). Mechanisms, prevention, and treatment of atrial fibrillation after cardiac surgery. J. Am. Coll. Cardiol..

[2] Maisel W.H., Rawn J.D., Stevenson W.G. (2001). Atrial fibrillation after cardiac surgery. Ann. Intern. Med..

[3] Bramer S., van Straten A.H., Soliman Hamad M.A., Berreklouw E., Martens E.J., Maessen J.G. (2010). The impact of new-onset postoperative atrial fibrillation on mortality after coronary artery bypass grafting. Ann. Thorac. Surg..

[4] Mathew J.P., Fontes M.L., Tudor I.C., Ramsay J., Duke P., Mazer C.D., Barash P.G., Hsu P.H., Mangano D.T. (2004). A multicenter risk index for atrial fibrillation after cardiac surgery. JAMA.

[5] El-Chami M.F., Kilgo P., Thourani V., Lattouf O.M., Delurgio D.B., Guyton R.A., Leon A.R., Puskas J.D. (2010). New-onset atrial fibrillation predicts long-term mortality after coronary artery bypass graft. J. Am. Coll. Cardiol..

[6] Mathew J.P., Parks R., Savino J.S., Friedman A.S., Koch C., Mangano D.T., Browner W.S. (1996). Atrial fibrillation following coronary artery bypass graft surgery: Predictors, outcomes, and resource utilization. MultiCenter Study of Perioperative Ischemia Research Group. JAMA.

[7] Lauer M.S., Eagle K.A., Buckley M.J., DeSanctis R.W. (1989). Atrial fibrillation following coronary artery bypass surgery. Prog. Cardiovasc. Dis..

[8] Mostafa A., El-Haddad M.A., Shenoy M., Tuliani T. (2012). Atrial fibrillation post cardiac bypass surgery. Avicenna J. Med..

[9] Dobrev D., Aguilar M., Heijman J., Guichard J.B., Nattel S. (2019). Postoperative atrial fibrillation: Mechanisms, manifestations and management. Nat. Rev. Cardiol..

[10] Ducceschi V., D’Andrea A., Liccardo B., Alfieri A., Sarubbi B., De Feo M., Santangelo L., Cotrufo M. (1999). Perioperative clinical predictors of atrial fibrillation occurrence following coronary artery surgery. Eur. J. Cardio-Thorac. Surg..

[11] van ‘t Hof A.W., Liem A., Suryapranata H., Hoorntje J.C., de Boer M.J., Zijlstra F. (1998). Angiographic assessment of myocardial reperfusion in patients treated with primary angioplasty for acute myocardial infarction: Myocardial blush grade. Zwolle Myocardial Infarction Study Group. Circulation.

[12] Gulec S., Atmaca Y., Kilickap M., Akyurek O., Aras O., Oral D. (2003). Angiographic assessment of myocardial perfusion in patients with isolated coronary artery ectasia. Am. J. Cardiol..

[13] Atmaca Y., Ozdemir A.O., Ozdol C., Oguz D., Gulec S., Kumbasar D., Erol C. (2005). Angiographic evaluation of myocardial perfusion in patients with syndrome, X. Am. J. Cardiol..

[14] Esenboga K., Baskovski E., Sahin E., Ozyuncu N., Tan T.S., Candemir B., Turhan S., Tutar E. (2020). Assessment of Myocardial Perfusion by Angiographic Methods in Tortuous Coronary Arteries. Angiology.

[15] Wijesurendra R.S., Liu A., Notaristefano F., Ntusi N.A.B., Karamitsos T.D., Bashir Y., Ginks M., Rajappan K., Betts T.R., Jerosch-Herold M. (2018). Myocardial Perfusion is Impaired and Relates to Cardiac Dysfunction in Patients with Atrial Fibrillation Both before and after Successful Catheter Ablation. J. Am. Heart Assoc..

[16] Mariscalco G., Engström K.G. (2009). Postoperative atrial fibrillation is associated with late mortality after coronary surgery, but not after valvular surgery. Ann. Thorac. Surg..

[17] Bramer S., van Straten A.H., Soliman Hamad M.A., Berreklouw E., van den Broek K.C., Maessen J.G. (2011). Body mass index predicts new-onset atrial fibrillation after cardiac surgery. Eur. J. Cardio-Thorac. Surg..

[18] Shingu Y., Kubota S., Wakasa S., Ooka T., Tachibana T., Matsui Y. (2012). Postoperative atrial fibrillation: Mechanism, prevention, and future perspective. Surg. Today.

[19] Omae T., Kanmura Y. (2012). Management of postoperative atrial fibrillation. J. Anesth..

[20] Magne J., Salerno B., Mohty D., Serena C., Rolle F., Piccardo A., Echahidi N., Le Guyader A., Aboyans V. (2019). Echocardiography is useful to predict postoperative atrial fibrillation in patients undergoing isolated coronary bypass surgery: A prospective study. Eur. Heart J. Acute Cardiovasc. Care.

[21] Osranek M., Fatema K., Qaddoura F., Al-Saileek A., Barnes M.E., Bailey K.R., Gersh B.J., Tsang T.S., Zehr K.J., Seward J.B. (2006). Left atrial volume predicts the risk of atrial fibrillation after cardiac surgery: A prospective study. J. Am. Coll. Cardiol..

[22] Miceli A., Romeo F., Glauber M., de Siena P.M., Caputo M., Angelini G.D. (2014). Preoperative anemia increases mortality and postoperative morbidity after cardiac surgery. J. Cardiothorac. Surg..

[23] Alameddine A.K., Visintainer P., Alimov V.K., Rousou J.A. (2014). Blood transfusion and the risk of atrial fibrillation after cardiac surgery. J. Card. Surg..

[24] Porto I., Hamilton-Craig C., Brancati M., Burzotta F., Galiuto L., Crea F. (2010). Angiographic assessment of microvascular perfusion--myocardial blush in clinical practice. Am. Heart J..

[25] Atmaca Y., Duzen V., Ozdol C., Altin T., Tulunay C., Ertas F., Erol C. (2008). Total blush score: A new index for the assessment of microvascular perfusion in idiopathic dilated cardiomyopathy. Coron. Artery Dis..

[26] Parwani A.S., Boldt L.H., Huemer M., Wutzler A., Blaschke D., Rolf S., Möckel M., Haverkamp W. (2013). Atrial fibrillation-induced cardiac troponin I release. Int. J. Cardiol..

[27] Range F.T., Schäfers M., Acil T., Schäfers K.P., Kies P., Paul M., Hermann S., Brisse B., Breithardt G., Schober O. (2007). Impaired myocardial perfusion and perfusion reserve associated with increased coronary resistance in persistent idiopathic atrial fibrillation. Eur. Heart J..

[28] Frustaci A., Chimenti C., Bellocci F., Morgante E., Russo M.A., Maseri A. (1997). Histological substrate of atrial biopsies in patients with lone atrial fibrillation. Circulation.

[29] Boldt A., Wetzel U., Lauschke J., Weigl J., Gummert J., Hindricks G., Kottkamp H., Dhein S. (2004). Fibrosis in left atrial tissue of patients with atrial fibrillation with and without underlying mitral valve disease. Heart.

[30] Skalidis E.I., Hamilos M.I., Karalis I.K., Chlouverakis G., Kochiadakis G.E., Vardas P.E. (2008). Isolated atrial microvascular dysfunction in patients with lone recurrent atrial fibrillation. J. Am. Coll. Cardiol..

[31] Wijesurendra R.S., Casadei B. (2015). Atrial fibrillation: Effects beyond the atrium?. Cardiovasc. Res..

